# A MicroRNA-Based Method for High-Viremia Detection—A New Approach on a Romanian Lot of Chronically Infected Patients with Hepatitis B Virus

**DOI:** 10.3390/diagnostics13223425

**Published:** 2023-11-10

**Authors:** Marina Manea, Dimitri Apostol, Ileana Constantinescu

**Affiliations:** 1 Deparment of Immunology and Transplant Immunology, University of Medicine and Pharmacy “Carol Davila”, 020021 Bucharest, Romania; 2 Centre of Immunogenetics and Virology, Fundeni Clinical Institute, 022328 Bucharest, Romania

**Keywords:** hepatitis B virus, microRNA, patient assessment, MiR-122

## Abstract

The HBV (hepatitis B virus) infection is intended for elimination, but evaluating patients is both costly and insufficiently applied in several countries. An expensive analysis in Romania is HBV-DNA quantification, with a limited prognostic potential. Our study intended to find new predictors for high viremia in HBV patients, using molecules involved in the multiple assessment of various HBV complications, such as microRNAs. A total of 61 subjects (48 patients with chronic HBV infection and 13 healthy subjects) were generally evaluated. Using a RT-PCR method, with a 2^−ΔΔCT^ algorithm, we detected the expressions of miR-122 and miR-146a in 33 subjects. MiR-21 was the internal control. The results were analyzed with the R 4.2.2. software. Kruskal–Wallis’s comparisons, Spearman correlations, and several logistic regression methods were applied. The median age of the patients was over 40 years. Without microRNAs, we could not obtain a good prediction formula. The combination of miR-122 and age proved to be the best prediction method for high viremia, with an AUC of 0.827, and a sensitivity of 89.5%. This is the first study which included age and miR-122 as independent predictors for high viremia in Romanian HBV-positive patients. MiR-122 is a new potential biomarker in the evaluation of Romanian patients.

## 1. Introduction

Infection with hepatitis B virus (HBV) is distributed in several countries around the world, especially in Asian and African areas [1]. Endemic HBV infection is a fact in regions of the European continent. However, the surveillance of infections with HBV across Europe might be an issue because of inappropriate measures of follow-up [2].

An interaction is known to occur between proteoglycans situated in the host’s cells and HBV (a member of Hepadnaviridae). Studies have depicted the relaxed circular DNA (rcDNA) as the previous in-line step in the formation process of the ovalently closed circular DNA (cccDNA). The viral transcription continues next. Studies have shown that several viral proteins are produced like this. Scholars also consider that next comes another step of reverse transcription, followed by the nucleocapsid’s encapsidation and its hepatocyte release. The proteins resulting from the replication phase activate the innate and adaptive immune responses. This leads to the enhancement of several pathways, as molecular elements involved in the chronic persistence of HBV and the development of complications such as fibrosis, autophagy, or HCC (hepatocellular carcinoma) [3]. Therefore, chronic infection with HBV might unfavorably complicate itself. The natural history of this disease is affected by several environmental, host-related, and virus-related factors. Some of those are the quantity of viral DNA, the persistence of HBV-DNA, and the viral genotype. Factors depending on several host traits also contribute to the evolution of chronic HBV infection (age and sex included) [4]. 

Researchers have used the presence of HBeAg, the levels of HBV-DNA, HBsAg, and alanine aminotransferase (ALT) to illustrate the stages of chronic HBV [5,6,7]. The degree of liver damage is also used in staging [5,6]. Therefore, the guideline of the European Association for the Study of the Liver (EASL) outlines the categories of chronic HBV by the presence or absence of HBeAg, together with the status of the illness (chronic infection or chronic hepatitis). According to EASL, chronic HBV hepatitis is mostly different from chronic HBV infection because of the high levels of HBV-DNA and ALT. Another category of patients is represented by those with occult HBV infection. HBsAg lacks in their sera [5]. Other guidelines have a similar classification of the main stages of chronic HBV [6,7]. All the international recommendations agree on the fact that chronic HBV patients should be assessed for the presence of severe complications such as cirrhosis, fibrosis, and HCC [5,6,7]. There are numerous surveillance strategies, but researchers have proved that non-invasive testing does not generally have the same diagnostic and prognostic accuracy as liver biopsy in establishing the degree of liver damage [8]. Several associations of biochemical and hematological tests were developed to improve the capacity of prediction for serious complications such as cirrhosis and HCC. Thus, ALT, AFP (alpha-fetoprotein) and platelet count were recently evaluated in a prognosis score for HCC in CHB (chronic hepatitis B) patients [9]. Another study shows that ALT, AFP, age, and liver stiffness are part of an evaluation score for liver damage in CHB with normal ALT levels [10]. RDW (red cell distribution width), together with other cellular ratios, was also used in predicting patient survival in CHB subjects with cirrhosis [11]. However, the best biomarker strategy for the prognosis of chronic HBV infection is still under debate.

MicroRNAs are some of the promising, new biomarkers studied in the grading and prognosis of CHB [12,13]. These are noncoding molecules, formed after a nuclear transcription phase and two cytoplasmic processing stages, mediated by DROSHA (the acronym comes from Drosha Ribonuclease III, one of the proteins of this complex) and other ribonucleases (called “Dicer”). The final, mature miRNAs modulate gene expression. Several strategies for microRNA regulation are currently under observation. The expressions of miRNAs are believed to involve epigenetic regulators and transcription factors currently studied in cancer treatment. This is because of the proved relationship between miRNAs and oncogenic pathways [14]. 

However, noncoding molecules are also known for their influence on the progression of HBV. Several microRNAs have been associated with the viral cycle, and with the evolution of chronic HBV infection (together with its complications) [15]. MiR-122 and miR-146a are some of the most frequently encountered microRNAs related to the fibrotic processes. In chronic HBV, the expressions of miR-122 and miR-146a are believed to be one upregulated and the other, respectively, downregulated [16]. Some authors find miR-146a as a proper biomarker associated with the progression of fibrosis in CHB [16,17], while others choose miR-122 as a prognostic biomarker of fibrosis [13,18]. Several correlations were found between miR-122 and the Child–Pugh scores in patients with cirrhosis [18]. Researchers have studied the accuracy of a new diagnostic panel for hepatocellular carcinoma (HCC), which was identified using miR-122, miR-16 and AFP [19]. MiR-122 was also studied in another combination panel for the diagnosis of HCC related to hepatitis C virus (HCV), together with other microRNAs. The results proved that microRNAs could replace the traditional AFP (alpha-fetoprotein) test [20]. Other microRNAs (miR-21-5p, miR-155-5p, miR-199a-5p) have also provided alternatives for AFP in the diagnosis of some HCC patients [21]. On the other hand, miR-146a was related to cancer progression and metastasis because of its interactions with pathways linked to TNF-α (tumor necrosis factor-alpha) and TGF-β (transforming growth factor-beta) [22]. To our knowledge, until now, no evaluation of costs has been made for microRNA panels in HBV. However, researchers have proved that, for testicular germ cell tumors, the use of some microRNAs is more cost-effective in the United States of America (USA) than several computer tomograph scans [23]. Therefore, detecting noncoding molecules in a patient’s serum might be a cost-effective procedure. This is because of the high versatility of these molecules and the numerous possibilities of using them in different prognostic strategies at the same time. However, this financial benefit could only arise if specific microRNAs are identified and used for both diagnosis and infection staging. At the time being, scientists have not yet found the precise microRNAs with high accuracy in staging the HBV infection. Several studies indicated that miR-122 might be a potential candidate for this [18].

Research has shown a current association between the high level of HBV-DNA and the unfavorable prognosis in CHB patients [5,6]. The level of HBV-DNA might be important in staging the HBV patients, and in evaluating their prognosis, but viremia detection might be a cost-demanding action. There is also a complex debate on how to classify the chronically infected HBV patients according to their HBV-DNA level. As presented in the current guidelines, the same categories of HBV-positive patients might have different levels of HBV-DNA [5]. 

Therefore, we decided to focus our research on the investigation of a potential connection between molecular, biochemical, and hematological parameters and the level of viremia. Our purpose was to find new predictor factors for the increase in the HBV-DNA level in chronically infected subjects using miR-122 and miR-146. This might be a new test for prognosis assessment in HBV patients. To our knowledge, this purpose is unique among the studies performed on Romanian subjects. Our discoveries intended to bring a promising perspective into the utility of microRNAs as diagnostic and prognostic biomarkers in patients with high viremia.

## 2. Materials and Methods

### 2.1. Patient Selection

Our study was carried out on 61 Romanian subjects, divided in two categories. A total of 48 were chronically HBV-infected patients (test lot), while 13 were healthy subjects (control lot). All patients from the test lot were HBeAg negative, and they were not under treatment either at the moment or before their enrollment. Both categories were selected without any discrimination between the sexes, and they were all responsible adults (over 18 years old). The period of HBsAg positivity (more than 6 months) was used for establishing the chronic HBV status. We did not enroll subjects with known associated coinfections (such as viral hepatitis C, viral hepatitis D, or COVID-19) or any documented cause of inflammation (such as myocardial infarction, diabetes, autoimmune diseases, or stroke). We also did not include pregnant women, or lactating mothers. The samples were obtained between 2020 and 2023 from the patients who were health assessed at Fundeni Clinical Institute, in Bucharest, Romania. Written consent was obtained from the enrolled subjects, and the entire study was performed after the approval of the Ethical Council of Fundeni Clinical Institute. We also paid attention to the Declaration of Helsinki. Venous blood sampling was performed on all the included subjects on heparin vacutainers (for hematological and molecular testing) and non-anticoagulant vacutainers (for serology and biochemical analysis). The resulting sera were kept at −80 °C in a specially designed storage area for molecular testing and at −20 °C for the usual serology testing. MicroRNA expressions of miR-122 and of miR-146a were assessed in 33 of the included subjects (27 patients and 6 controls).

### 2.2. Parameter Acquisition

Data regarding the subjects’ current diagnosis and clinical parameters were collected from the existing records deposited at Fundeni Clinical Institute. The levels of the biochemical parameters were determined using the Versacell V2 machine (Siemens Healthineers GmbH, Erlangen, Germany). The subjects’ hemograms were performed using SYSMEX-XN-1000-05 (Sysmex Europe, Norderstedt, Germany). The viral antigens were detected with ADVIA CENTAUR XPT_2 (Siemens Healthineers GmbH, Erlangen, Germany). The quantification of HBV-DNA was investigated with BOSPHORE VHB QUANTIFICATION KIT (Anatolia geneworks, Istanbul, Turkey).

### 2.3. MicroRNA Detection

The detection of miR-122 and miR-146a was performed using a Thermo Fisher kit based on a magnetic bead protocol. The first step of this process consisted of the extraction of total RNA from 100 µL of sera. This was achieved after the digestion of the treated samples at 65 °C, followed by both a lysis step and a binding phase of the total RNA. The binding phase consisted in an attachment process of the total RNA to some specially designed beads provided by the manufacturer. The next step was the reverse transcription phase carried after the following cycle: 30 min at 16 °C, 30 min at 42 °C, 5 min at 85 °C, and a cycle finish at 4 °C. The last step in obtaining the miRNA quantification was a real-time PCR amplification performed on a thermo-cycler set with the following mode: 1 cycle of 10 min at 95 °C for the activation of the enzyme, and 40 cycles consisting in a denaturation phase for 15 s at 95 °C, and an annealing/extension phase for 60 s at 60 °C. The relative expressions of miR-122 and miR-146a were determined using a 2^−ΔΔCT^ method. The expression of miR-21 was considered the internal control of the reactions. For every mature microRNA, we used a primer. The cDNA (cyclic DNA) resulted was then quantified by quantitative RT-PCR (qRT-PCR) using Applied Biosystems 7300 (Applied Biosystems, Thermo Fisher Scientific, San Francisco, California, USA). Each primer was complementary to the mature sequence depicted in Table 1. All reagents were supplied by Applied Biosystems, Thermo Fisher Scientific, San Franscisco, CA, USA.

### 2.4. Statistical Analysis

The statistical analysis was performed using R 4.2.2 software [24]. All variables were presented as median (and interquartile) or percentages (where it was needed). The normality of parameters was investigated following the skewness and kurtosis of data, together with the Shapiro test for normality and the graphical representation of data distribution. The intergroup differences were evaluated by the Mann–Whitney and Kruskal–Wallis tests. A pairwise post hoc comparison was performed using Dunn’s test. Univariate, multivariate logistic regression tests and a backwise selection using Akaike Information Criteria were performed to identify the parameters that best predicted the high HBV-DNA level (defined as being over 2000 IU/mL). Correlations were assessed with a Spearman correlation method. ROC (receiver-operator curves) were performed to detect the best parameters for identification of high levels of HBV-DNA.

## 3. Results

### 3.1. Subjects’ Characteristics

**Figure 1:**
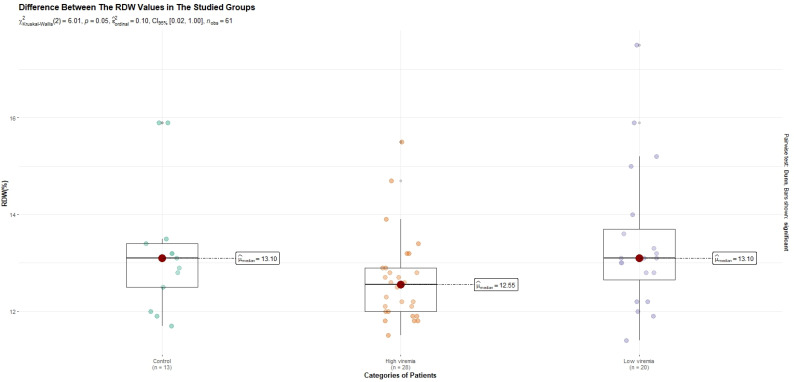
Intergroup analysis of RDW (%) with Holm adjustment.

The 61 analyzed subjects had a median age of 46 years and 44.3% of them were males. Twenty-eight patients had high viremia (defined as a HBV-DNA value over 2000 IU/mL). Low HBV-DNA levels (under 2000 IU/mL) were detected in twenty patients. We compared the levels of the studied parameters between the three groups of subjects included in our study (those with high viremia, those with low-viremia and healthy subjects). A *p*-value under 0.05 was considered significant. We discovered that only ALT and RDW were differently expressed between all three groups. Patients with high viremia tended to have higher ALT levels (*p* = 0.001) and lower RDW percentages (*p* = 0.049) in comparison to those with low viremia. The *p*-values were summarized in Table 2. However, after a Holm-based adjustment of the *p*-values obtained from intergroup comparisons of both RDW percentages and ALT serum levels, we observed that the RDW percentages were no longer significantly different between the categories of subjects (this is shown in Figure 1). HBsAg was positive in all HBV patients.

### 3.2. Logistic Regression Analysis

The predictors for high viremia were retrieved through logistic regression. For the univariate analysis, a *p*-value under 0.2 was considered significant because those results were later used in multivariate analysis (univariate analysis was only a preliminary test). The predictors were age (measured in years), total bilirubin serum levels (mg/dL) and RDW values (established in percentages). The results of the univariate analysis are shown in Table 3. Akaike Information Criterion (stepwise backward logistic regression) and multivariate logistic regression analysis were performed to obtain the best prediction model for high viremia. A *p*-value under 0.05 was considered significant. ROC (receiver-operator curves) were constructed for the models with the lowest *p*-values. The combination of the variables age (years) and RDW (%) was considered the best predictor of high viremia (with a *p*-value under 0.001). However, the AUC (area under the curve) for this model was low (0.716), with a sensitivity of 78.6% and a specificity of 66.7% (the results are depicted in Table S1 and Figure S1).

### 3.3. Characteristics of the microRNA Subgroup Subjects

**Figure 2:**
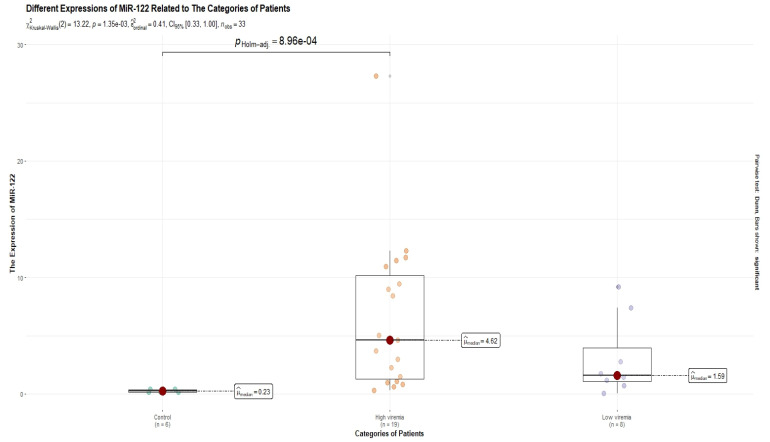
The differences in the expression levels of miR-122 between the groups. The only statistically significant difference was observed between the control lot and the high-viremia lot (*p* < 0.001). The scientific notation 1.35 × 10^−3^ is equivalent to 0.00135. The scientific notation 8.96 × 10^−4^ is equivalent to 0.000896.

**Figure 3:**
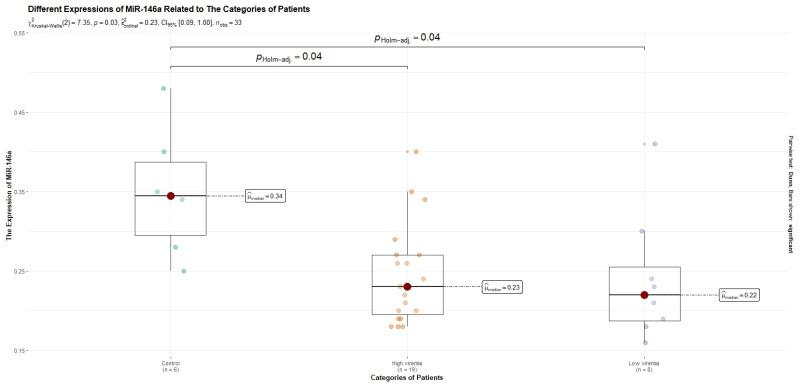
The differences in the expression levels of miR-146a between the groups. The statistically significant differences were observed between the control lot and the high-viremia lot (*p* < 0.05), and between the healthy controls and the low-viremia subjects (*p* < 0.05). There were no statistically significant differences (*p* > 0.05) between the patients with high viremia and those with low viremia.

A subgroup of 33 subjects was randomly selected from a total of 61 patients. The median age was 46 years, and 48.5% of the selected subjects were males. Eight patients had low viremia (<2000 IU/mL). High HBV-DNA levels (>2000 IU/mL) were detected in 19 subjects. In the HBV-positive patients, by comparison to the healthy controls, miR-122 was upregulated (fold change = 12.04), while miR-146a was downregulated (fold change = −1.44). Patients with high HBV-DNA levels had a lower expression of miR-122 (fold change = 11.15), as compared with those with low viremia (fold change = 19.29). On the other hand, the expression of miR-146a was higher in those with high viremia (fold change = −1.23), as compared with those with low viremia (fold change = −1.69). However, these differences were not statistically significant after a Holm adjustment of the *p* values. As compared to the control lot, the ALT levels and the expression levels of miR-122 were higher (*p* < 0.05), whereas the expression levels of miR-146a were lower (*p* < 0.05). The characteristics of these subjects were depicted in Table 4. After a Holm adjustment of the *p*-values, the expression of miR-146a also remained significantly different between the HBV-infected patients with low-level viremia and the control lot (*p* < 0.05). Therefore, both patients with high and low viremia presented a lower miR-146a expression by comparison to the control lot. These differences are shown in Figure 2 and Figure 3. Overall, there were no significant correlations between the values of the variables taken into consideration.

### 3.4. Logistic Regression Analysis of the microRNA Subgroup

Univariate logistic regression analysis indicated age (years), miR-122 and miR-146a as potentially significant prediction factors for high viremia (see Table 5). Stepwise backward logistic analysis and multivariate logistic analysis were performed to depict the variables associated with high viremia (see Table S2, Figures S2–S4). For the logistic analysis, a *p*-value under 0.2 was considered significant, because we only wanted to set a trend that would be checked in future studies. The number of patients also contributed to our decision. A final model, based on the expression level of miR-122, and age (measured in years) was the best option. The mathematical formula of this new model associated with high levels of HBV-DNA is:

Model = 1 − 1/(1 + exp(2.26207351542858 + (Age (years) × (−0.0593343750917939)) + (miR-122 × 0.240188447301305)))

**Figure 4:**
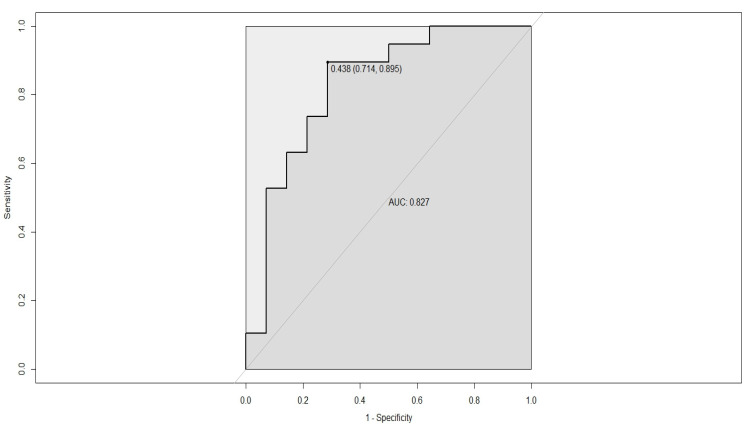
ROC curve for a logistic model based on two variables: age (years) and the expression of miR-122; AUC-aria under the curve. The numbers listed above AUC represent the best threshold, and the calculated specificity and sensitivity (the last two values were placed in parenthesis). The model had the highest AUC curve. This combination of variables was also chosen by stepwise backward logistic regression as a possible predictor of high viremia.

The AUC of this model was 0.827, with a specificity of 71.4% and a sensitivity of 89.5% (Figure 4). 

## 4. Discussion

The HBV-DNA load is an important assay for chronically infected people. Guidelines recommend viremia detection for the selection of the treatment protocol and the follow-up strategy of the patients [5]. In a recent study performed on 106 subjects, the authors attempted to use the viral load as a predictor for moderate inflammation in CHB patients with negative HBeAg [25]. The prognostic value of high HBV-DNA has also been emphasized in a recent study on Caucasian, HBeAg-negative CHB patients [26]. However, HBV-DNA remains an expensive analysis, especially in Eastern European countries. Another problem in using viral loads as predictors for the patient’s outcome arises from the fluctuating capability of HBV-DNA within the different stages of the chronic infection [5]. This is because of the activation of various pathways related to the presence of HBV inside the host’s cells. It has been already shown that viral proteins (such as HBx) stimulate viral replication through various molecular factors such as RLF [27] (“Rearranged L-myc fusion protein” [28]), CDT1 [27] (“Cdc10-dependent transcript 1 protein” [29]) or PCAF [27] (“P300/CBP-Associated Factor” [30]). Some other viral components play an important role in cell cycle regulation, DNA repair, apoptosis, and oxidative stress. These interactions between viral particles and cellular components of the host are encountered in the evolution of the chronic infection and eventually lead to HCC [27]. 

The idea of using molecular biomarkers instead of traditional ones for the patient’s follow-up arises from the promising versatility of microRNAs during the development of chronic HBV infection. MiR-55-5p, miR-193b-5p, miR-200b-3p and miR-3175 were recently studied as molecules involved in cellular pathways such as PI3K/AKT [31] (“phosphatidylinositol-3-kinase” [32]/“protein kinase B” [33]), Ras [31] (“Rat sarcoma” [34]), MAPK [31] (“mitogen-activated protein kinase” [35]). Such pathways were related to the development of acute-on-chronic liver failure associated with HBV [31]. HBx downregulated the cellular expression of miR-122 in studies involving cell cultures performed on samples collected from HBV-HCC patients [36]. MiR-122 and miR-146a were related to the development of fibrosis in chronic HBV infection [16]. Newly developed studies focus on the importance of microRNAs in many molecular pathways related to HBV as future biomarkers in diagnosis, in prognosis, and as promising treatment options [37]. Although scientists have not yet discovered the best microRNA formula for the diagnosis and the evaluation of chronically infected HBV patients, many studies are focused on the potential of miR-122 [13,15,16,18,19] and miR-146a [16,17]. 

Finding a versatile molecular biomarker with both diagnostic and prognostic capabilities could be both cost-beneficial and time-effective. No one has performed until now an economic analysis of the impact of microRNAs as diagnostic and prognostic biomarkers for Romanian patients. However, because the current evaluation protocols for HBV involve multiple analysis (viremia, levels of HBsAg, HBeAg, sometimes even AFP, computer tomographs and liver biopsy) [5], one biomarker is needed to reduce at least some of these costs. The obvious choice is the study of molecules which could potentially replace some of the above-mentioned tests. This could reduce costs because of the reduction in the numbers of assays needed for one patient. Although the costs of microRNAs seem comparable to viremia detection, or a little more expensive [38,39], they could have the potential to replace some of the evaluation assays [20,21,23]. The cost of a microRNA test is estimated at 30 USD [38], while a viremia test is estimated at an average of 15 USD, but it can reach 100 USD [39]. Adding the costs of computer tomographs (with an average of approximately 100 USD) [40], together with those of liver biopsy (with an average of approximately 224 USD) [41], the complete evaluation of one HBV patient surpasses the price of a single microRNA test. However, this economic benefit should be further assessed, together with the potential of microRNAs in replacing viremia, liver biopsy or computer tomographs. Therefore, our research performed the first steps of such complex studies. Our article focused on finding new predictor factors for high HBV-DNA using the expressions of miR-122 and miR-146a. The median age of the included subjects was 46 years, while the median age of those with high viremia was 41 years. Patients with low viremia had a similar median age. This suggested the fact that most ages were situated in the fourth decade. Older age of CHB patients with detectable viremia has been globally observed, mostly because of diagnostic faults and insufficient monitoring [42]. This age tendency of Romanian chronically infected HBV patients has also been observed in previous studies on larger cohorts [43,44,45]. 

Our research firstly retrieved two parameters (age-expressed in years and RDW as %) as potential predictors of high HBV-DNA, with a *p*-value of <0.05. These results were confirmed both in univariate and multivariate analyses. This is, to our knowledge, the first study that finds such an association of predictors for high-level viremia. However, the combination of these two variables could be improved. As it was earlier depicted in our results, such a model might only have an AUC of 0.716, with the best threshold of 0.446, a low specificity (66.7), and a low sensitivity (78.6%). Adding new predictors could increase the specificity and the sensitivity of the formula. In another study, age seemed to be related to the activity of T lymphocytes in CHB patients [46]. The prognostic potential of the patients’ ages was also analyzed on a large cohort for risk quantification of hepatocellular carcinoma [47]. The age variable is also part of prognostic scores which assess the degree of fibrosis in HBV patients (such as FIB4-fibrosis score 4), but with low sensitivity and low specificity in comparison with the invasive techniques of liver evaluation [48]. RDW is, on the other hand, another possible prognostic factor for HBV complications, such as decompensated cirrhosis [11]. High HBV-DNA levels have also been linked to the stages and the prognosis of the HBV complications [49,50]. This might be the first explanation of the association between RDW, age and high HBV-DNA.

We tried to find a molecular explanation for the connection between the biochemical, clinical characteristics, and the variations in the viral loads. Therefore, we took into consideration microRNAs, mostly because of their known relations with chronic HBV infection [15,16,17,18]. We identified differences between the serum levels of both miR-122 and miR-146a in chronic HBV patients with high viremia, in comparison with normal controls (*p* < 0.001, and, respectively, *p* < 0.04). A possible connection between viremia and serum levels of miR-122 was previously documented. A study, for instance, showed a positive correlation between the level of miR-122 and the viral load in HBV patients [13]. Other researchers linked the expression of miR-122 with the positive virological response encountered in patients with CHB, treated with nucleos(t)ide analogs (NAs) [51]. In a large study performed on over 400 patients with chronic hepatitis B and cirrhosis, Jin et al. discovered that the expression of miR-122 could differentiate those with high viremia from those with low viral loads. This microRNA was upregulated in CHB patients and inhibited in cirrhotic subjects. The same authors found that miR-146a was upregulated in CHB patients [52]. In another study performed on Romanian patients with HCC, the expression of miR-122 was upregulated in HBV carriers [53]. On the other hand, miR-146a is mostly studied in immunology-related studies. An example is the research connected with the inflammation process, generated and amplified by the presence of HBV proteins [54]. Another study shows that miR-146a acts on the intrinsic, immunology-related activities by regulating RIG-I [55]. However, research containing miR-146a, made on human HBV subjects, is scarcely encountered in the literature. However, scientists have proved a possible prediction capability of this molecular biomarker in 282 CHB patients [56]. Our research showed that miR-122 was upregulated and miR-146a was downregulated in HBV patients with high viremia, by comparison to healthy controls. However, the differences between the expressions of these two microRNAs encountered in high-viremia and low-viremia patients were without statistical meaning. This might be because there are other molecular features (such as other microRNAs) that also interfere in the replication process in chronically infected HBV patients. Such connections might be of future interest for new research strategies. Still, this study is the first that analyzed the expression of both miR-146a and miR-122 in Romanian HBV patients, associating them with high viral loads.

After performing univariate and multivariate logistic regression analyses, age (measured in years) and miR-122 were the only predictors linked, in a significant, statistical manner, to high viremia. Therefore, a new formula was established for identifying high-viremia patients, using the expression of miR-122 and age. The ROC curve for this formula had an AUC of 0.827, with a specificity of 71.4%, and a sensitivity of 89.5%. This AUC was better than those obtained for age only (AUC = 0.727), and for miR-122 only (AUC = 0.806), taken as single, independent predictors for high viremia. In another study performed on Romanian patients with HBV-associated HCC, age influenced the expression of microRNAs [53]. In our study, no significant correlation was found between age and the expressions of miR-122 and miR-146a. The good AUC obtained for the combination of age and miR-122 as opposed to the AUC found for the association between age and RDW (AUC = 0.716) shows that combining molecular biomarkers to other predictors for high viremia is the best strategy for identifying high HBV-DNA.

This research shows that by taking only clinical, biochemical, and hematological features as possible predictors for high viremia in adult, chronic HBV patients, the results are prone to the incorrect identification of subjects. However, if a molecular feature is included in the equation, the obtained results are improved. This is probably because of the complexity of molecular pathways that HBV activates [27,31,36,37] that cannot be entirely reflected in the variability of traditional markers.

However, our research has several limitations. Firstly, the number of included subjects is small because this was a preliminary study. This could influence to a certain degree the expression of microRNAs. Secondly, our microRNA-based prediction method does not have the best specificity. We intend to improve our current preliminary research by further studies performed on a larger scale and by adding various other microRNAs, to better differentiate high-viremia patients from low-viremia patients. 

Still, this study provides, for the first time, a new start-up model for the assessment of chronically HBV-infected Romanians. This is a promising first step in the establishment of a new microRNA evaluation strategy for HBV-positive patients. 

## 5. Conclusions

Investigating microRNAs in Romanian HBV patients is of current interest, especially because research on this topic in this country is scarce. Establishing new assessment methods for chronically infected HBV subjects is also important in Eastern European countries, where the methods used for the diagnosis and prognosis of these patients are less developed, and where there might be cost-related difficulties. Our study shows, for the first time, that an association between age and miR-122 could be used as a new effective method for detecting high-level viremia in Romanian chronically infected HBV patients. This is a new step in using microRNAs in the assessment strategies for the evolution of the HBV infection.

## Figures and Tables

**Table 1:** The investigated microRNAs. For each microRNA, we used a primer complementary to the mature sequence provided below.

Assay Name	Assay ID	Mature miRNA Sequence	Chromosome Location
hsa-miR-21	000397	UAGCUUAUCAGACUGAUGUUGA	Chr.17
hsa-miR-122	002245	UGGAGUGUGACAAUGGUGUUUG	Chr.18
hsa-miR-146a	000468	UGAGAACUGAAUUCCAUGGGUU	Chr.5

**Table 2:** Clinical characteristics of the studied subjects.

Variable	Total [Interquartile] (*n* = 61)	High Viremia [Interquartile] (*n* = 28)	Low Viremia [Interquartile] (*n* = 20)	Controls [Interquartile] (*n* = 13)	*p*-Value **
Age (years)	46 (35, 56)	41 (34.8, 49)	44.5 (37.2, 59.0)	52 (49, 60)	0.064
Sex—male (%)	27 (44.3)	11 (39.3)	12 (60.0)	4 (30.8)	0.197
ALT * (U/L)	25 (19, 31)	29.5 (24.5, 32.5)	25.5 (18.8, 35.8)	20 (16, 22)	0.001
AST * (U/L)	23 (21, 26)	24 (22, 26)	23 (21.0, 29.5)	22 (18, 23)	0.113
Total bilirubin (mg/dL)	0.6 (0.5, 0.8)	0.7 (0.6, 0.8)	0.5 (0.5, 0.7)	0.6 (0.4, 0.6)	0.088
RDW * (%)	12.8 (12.1, 13.2)	12.6 (12.0, 12.9)	13.1 (12.7, 13.7)	13.1 (12.5, 13.4)	0.049
Leucocyte count (×10^3^ µL)	6.5 (5.7, 7.7)	6.1 (5.5, 7.3)	6.9 (5.9, 7.5)	6.1 (5.8, 7.9)	0.486
Platelet count (×10^3^ µL)	247 (206, 305)	258.5 (209.5, 289.2)	230.5 (185.2, 276.5)	274 (238, 314)	0.132

* ALT—alanine aminotransferase, AST—aspartate aminotransferase, and RDW—red cell width; ** *p*-value < 0.05 was considered significant.

**Table 3:** The results of univariate logistic analysis.

Variables	OR *	95% CI *	*p*-Value **
Age (years)	0.96	0.91, 1.00	0.044
Sex—male (%)	0.69	0.24, 1.9	0.5
ALT * (U/L)	1.03	0.99, 1.07	0.2
AST * (U/L)	1.0	0.92, 1.07	0.9
Total Bilirubin (mg/dL)	6.4	1.17, 48.1	0.047
RDW * (%)	0.57	0.31, 0.92	0.039
Leucocyte count (×10^3^ µL)	0.91	0.65, 1.24	0.6
Platelet count (×10^3^ µL)	1.00	0.99, 1.01	0.8

* OR—odds ratio, CI—confidence interval, ALT—alanine aminotransferase, AST—aspartate aminotransferase, and RDW—red cell width; ** *p* < 0.2 was considered significant.

**Table 4:** The characteristics of the microRNA subgroup.

Variable	Total [Interquartile] (*n* = 33)	Low Viremia [Interquartile] (*n* = 8)	High Viremia [Interquartile] (*n* = 19)	Controls [Interquartile] (*n* = 6)	*p*-Value **
Age (years)	46 (38, 59)	49.5 (45.2, 60.0)	42 (36.0, 50.5)	56 (52.0, 64.5)	0.076
Sex—male (%)	16 (48.5)	5 (62.5)	9 (47.4)	2 (33.3)	0.551
ALT * (U/L)	27 (21, 34)	26.5 (22.5, 49.5)	31 (26.5, 37.5)	15.5 (14.2, 19.8)	0.005
AST * (U/L)	23 (21, 27)	24.5 (22.0, 31.2)	24 (21.5, 26.0)	20 (17.5, 22.5)	0.195
Total Bilirubin (mg/dL)	0.6 [0.5, 0.8)	0.6 [0.5, 0.8)	0.7 [0.6, 0.8)	0.6 [0.4, 0.6)	0.275
RDW^*^ (%)	12.7 (12.0, 13.1)	13.1 (12.6, 13.1)	12.3 (12.1, 12.9)	12.6 (12.1, 12.9)	0.521
Leucocyte count (×10^3^ µL)	6.5 (5.8, 7.2)	6.9 (6.5, 7.4)	6.5 (5.7, 7.4)	5.9 (5.5, 6.10	0.223
Platelet count (×10^3^ µL)	261 (210, 293)	256.5 (200.5, 314.0]	261 (215.0, 280.5)	260 (240.0, 297.2)	0.956
miR-122	1.8 (0.6, 8.4)	1.6 (1.1, 3.9)	4.6 (1.3, 10.2)	0.2 (0.1, 0.4)	0.001
miR-146a	0.2 (0.2, 0.3)	0.2 (0.2, 0.3)	0.2 (0.2, 0.3)	0.3 (0.3, 0.4)	0.025

* ALT—alanine aminotransferase, AST—aspartate aminotransferase, and RDW—red cell width; ** *p*-value < 0.05 was considered significant.

**Table 5:** The results of the univariate logistic analysis on the microRNA subgroup.

Variables	OR *	95% CI *	*p*-Value **
Age (years)	0.93	0.87, 0.99	0.037
Sex—male (%)	0.90	0.22, 3.63	0.9
ALT * (U/L)	1.02	0.97, 1.09	0.4
AST * (U/L)	1.0	0.90, 1.12	>0.9
Total Bilirubin (mg/dL)	4.6	0.59, 69.9	0.2
RDW * (%)	0.98	0.32, 2.52	>0.9
Leucocyte count (×10^3^ µL)	1.11	0.75, 1.78	0.6
Platelet count (×10^3^ µL)	1.00	0.99, 1.01	0.8
miR-122	1.31	1.07, 1.76	0.03
miR-146a	0.00	0.00, 7.65	0.15

* OR—odds ratio, CI—confidence interval, ALT—alanine aminotransferase, AST—aspartate aminotransferase, and RDW—red cell width; ** *p* < 0.2 was considered significant.

## Data Availability

The data can be obtained from the corresponding author upon reasonable request.

## Supplementary Materials

The following supporting information can be downloaded at: https://www.mdpi.com/article/10.3390/diagnostics13223425/s1, Figure S1: ROC curves for the logistic models found on the entire lots. Figure S2: ROC curve for a logistic model based on the age (years) variable; Figure S3: ROC curve for a logistic model based on the expression of miR-122; Table S1: Models included in multivariate logistic analysis; Table S2: Multivariate logistic analysis on the models from microRNA subgroup.
