# Supplementary material for: A MicroRNA-Based Method for High-Viremia Detection—A New Approach on a Romanian Lot of Chronically Infected Patients with Hepatitis B Virus
Source: Diagnostics (Basel). 2023 Nov 10;13(22):3425. doi: 10.3390/diagnostics13223425 (PMC10670501; doi:10.3390/diagnostics13223425)
Supplement: Supplementary file 1 [file diagnostics-13-03425-s001.zip › Figure S1.pdf]

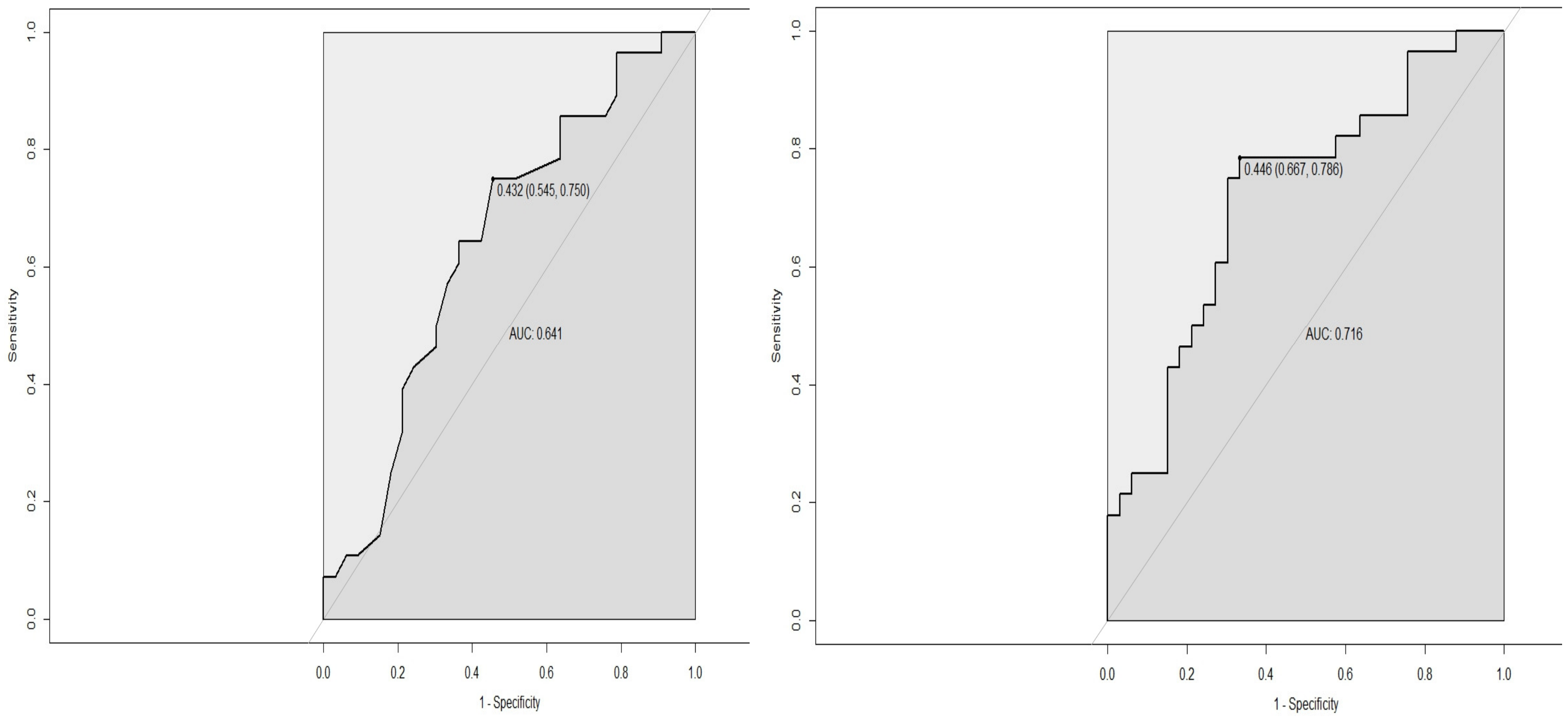

(**a**) (**b**)

Figure S1. ROC curves for the logistic models found on the entire lots. (**a**) ROC curve for a logistic model based on the age (years) variable; (**b**) ROC curve for a logistic model based on age (years) and RDW (%). AUC-aria under the curve. The numbers listed above AUC represent the best thresholds, and the calculated specificity and sensitivity (the last two values were placed in parenthesis). Both models used for high viremia prediction had low AUCs.
