# Supplementary material for: A MicroRNA-Based Method for High-Viremia Detection—A New Approach on a Romanian Lot of Chronically Infected Patients with Hepatitis B Virus
Source: Diagnostics (Basel). 2023 Nov 10;13(22):3425. doi: 10.3390/diagnostics13223425 (PMC10670501; doi:10.3390/diagnostics13223425)
Supplement: Supplementary file 1 [file diagnostics-13-03425-s001.zip › Figure S3.pdf]

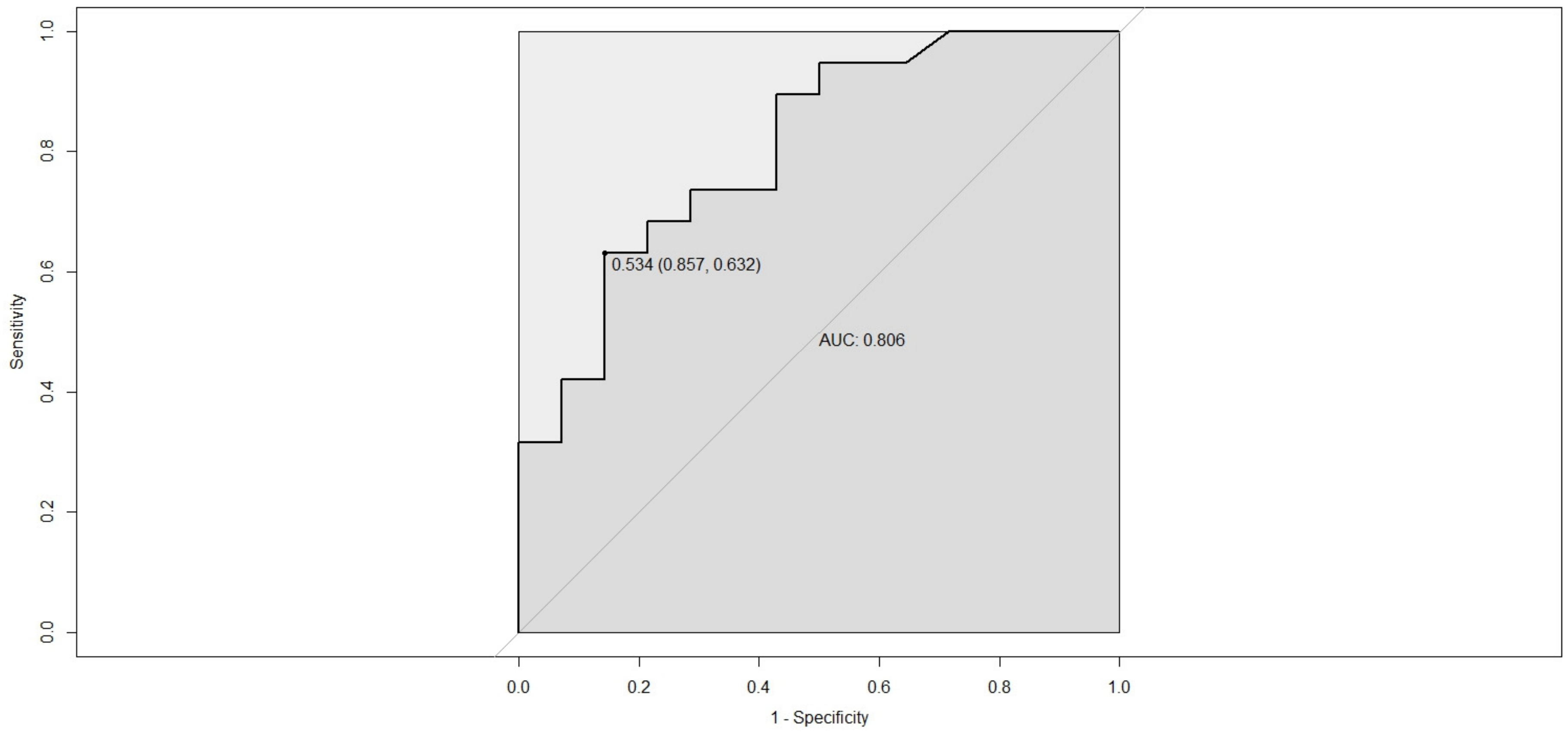

Figure S3. ROC curve for a logistic model based on the expression of miR-122; AUC-aria under the curve. The numbers listed above AUC represent the best threshold, and the calculated specificity and sensitivity (the last two values were placed in parenthesis). This model had an AUC lower than the final model.
