# Supplementary material for: A MicroRNA-Based Method for High-Viremia Detection—A New Approach on a Romanian Lot of Chronically Infected Patients with Hepatitis B Virus
Source: Diagnostics (Basel). 2023 Nov 10;13(22):3425. doi: 10.3390/diagnostics13223425 (PMC10670501; doi:10.3390/diagnostics13223425)
Supplement: Supplementary file 1 [file diagnostics-13-03425-s001.zip › Table S1.pdf]

Table S1. Models included in multivariate logistic analysis.

	Model 1			Model 2			Model 3		
**Estimates**	**Estimates**	**95% CI***	***p*-value****	**Estimates**	**95% CI***	***p*-value****	**Estimates**	**95% CI***	***p*-value****
(Intercept)	0.96	0.48-1.44	<0.001	2.35	1.03-3.67	<0.001	1.96	0.66-3.26	0.003
Age (years)	-0.01	-0.02-0.00	0.035	-0.01	-0.02-0.00	0.042			
RDW* (%)				0.11	-0.21--0.01	0.028	-0.12	-0.21--0.02	0.023

*RDW-red cell width, CI-confidence interval; ***p* < 0.2 was considered significant. The lowest *p*-values were obtained from models 1 and 2, so they were considered the best.
