# Supplementary material for: A MicroRNA-Based Method for High-Viremia Detection—A New Approach on a Romanian Lot of Chronically Infected Patients with Hepatitis B Virus
Source: Diagnostics (Basel). 2023 Nov 10;13(22):3425. doi: 10.3390/diagnostics13223425 (PMC10670501; doi:10.3390/diagnostics13223425)
Supplement: Supplementary file 1 [file diagnostics-13-03425-s001.zip › Table S2.pdf]

Table S2. Multivariate logistic analysis on the models from microRNA subgroup.

	Model 1			Model 2			Model 3		
**Estimates**	**Estimates**	**95% CI***	***p*-value****	**Estimates**	**95% CI***	***p*-value****	**Estimates**	**95% CI***	***p*-value****
(Intercept)	1.30	0.66-1.93	<0.001	0.98	0.28-1.67	0.006	0.42	0.21-0.62	<0.001
Age	-0.02	-0.03-0.00	0.021	-0.01	-0.02-0.00	0.1			
miR-122				0.03	0.00-0.06	0.065	0.04	0.01-0.06	0.013

* CI-confidence interval; ***p* < 0.2 was considered significant because of the small number of subjects.
